# Supplementary material for: The Capability of O-Acetyl-ADP-Ribose, an Epigenetic Metabolic Small Molecule, on Promoting the Further Spreading of Sir3 along the Telomeric Chromatin
Source: Genes (Basel). 2019 Jul 30;10(8):577. doi: 10.3390/genes10080577 (PMC6723988; doi:10.3390/genes10080577)
Supplement: Supplementary file 1 [file genes-10-00577-s001.pdf]

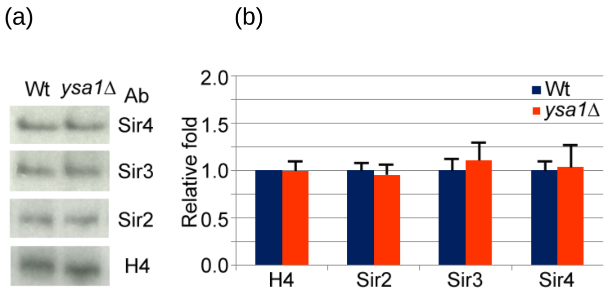

Figure S1. Relative amounts of Sir proteins in wild-type and *ysa1*-deletion strain cells. (a) Western blots of Sir4, Sir3, and Sir2 were detected in wild-type (Wt) and *ysa1*-deletion (*ysa1Δ*) strain cells. Histone 4 protein (H4) was used as an internal control and to normalize the signal. (b) Quantification of results from (a). Ab: antibody.

Table S1. List of strains used in this study

Name	Genotype	Source
W303/SF1	JRY2334, *Mat a ade2-1 can1-100 his3-11 leu2-3.112 trp1 ura3-1 GAL*	J. Rine
DMY2376	W303, *pep4Δ::LEU2*	A. Rudner
DMY3993	DMY2376, *Sir3-Myc13-Kam*	A. Rudner
LY060	DMY2376, *Sir3-Myc13-Kam Ysa1Δ::HGH*	This work
DMY3628	DMY2376, *Sir3-TAP-K.l-TRP1*	A. Rudner
LY059	DMY2376, *Sir3-TAP-K.l-TRP1 Ysa1Δ::HGH*	This work
DMY3810	DMY2376, *Sir3N*$_{(BAH1\text{-}214\ aa)}$*-TAP-K.l-TRP1*	G. Li
DMY3836	DMY2376, *Sir3C*$_{(215\text{-}978\ aa)}$*-TAP-K.l-TRP1*	Onishi et al., 2007
SF10	BJ5459, *Mat a ura3-52 trp1 lys2-801 leu2Δ1 his3Δ200 pep4ΔHIS prb1Δ1.6R can1 GAL*	E. Jones
DMY2364	SF10, pDM598 (pGAL-Sir3TAP)	Tanny et al., 2004
DMY2298	SF10, pDM654 (pGAL-TAP-Sir4), pDM641 (pGAL-HA-Sir2)	Liou et al., 2005
DMY3392	*HTA2-TAP-K.l.-TRP1*	Onishi et al., 2007

Table S2. Primers used in qRT-PCR analyses

Gene	Forward primer	Reverse primer
YAL068C	5'-TAACTTCAATCGCCGCTGGT-3'	5'-CACTGGAGATGGCTGGCTTT-3'
YAL067C	5'-GCCAATTATAGGGGTGCCGA-3'	5'-ACGACTCCCAACACACGTTT-3'
YAL065C	5'-GCTGCTGAGACAACTACCAGT-3'	5'-CGATTGCCAGCAATACGGTG-3'
YBR302C	5'-TGCGAATGGATGGGACGAAA-3'	5'-CTGCGGGATAATTGCGCTTC-3'
YBR301W	5'-CACCACCATGTTGACCGGTA-3'	5'-GAGCACTAGAGATGGCTGGC-3'
YBR299W	5'-AAAGGTCTGGCCCACATACG-3'	5'-TCCAGAAGAACCAGTCACGC-3'
YKL224C	5'-CCCCAGCCACTACCACTCTA-3'	5'-ACCGTCCTTGGATAGAGCAC-3'
YKL222C	5'-CCGCCTGACATTCCACTCAT-3'	5'-ATTTAGTGGAGCCAGGACGC-3'
YKL221W	5'-TGTTGGGCCATGTGGATACC-3'	5'-GGGCAACTAAGGCAAAAGCC-3'
TEL0.07K (ORA149 & ORA150)	5'-CATGACCAGTCCTCATTTCCATC-3'	5'-ACGTTTAGCTGAGTTTAACGGTG-3'
TEL0.6K (DM241 & DM242)	5'-CAGGCAGTCCTTTCTATTTC-3'	5'-GCTTGTTAACTCTCCGACAG-3'
YFR056C	5'-TTTCATTGTGGTGTCCCAAC-3'	5'-CGCCGTAGCATCCAAATAAT-3'

Actin<br>(JH301 & JH302)	5'-GCCTTCTACGTTTCCATCCA-3'	5'-GGCCAATCGATTCTCAAAA-3'
